# A Sphingolipidomic Profiling Approach for Comparing X-ray-Exposed and Unexposed HepG2 Cells

**DOI:** 10.3390/ijms241512364

**Published:** 2023-08-02

**Authors:** Martina Moggio, Bahar Faramarzi, Marianna Portaccio, Lorenzo Manti, Maria Lepore, Nadia Diano

**Affiliations:** 1 Department of Experimental Medicine, University of Campania “Luigi Vanvitelli”, 80138 Naples, Italy; martina.moggio@unicampania.it (M.M.); marianna.portaccio@unicampania.it (M.P.); maria.lepore@unicampania.it (M.L.); 2 Department of Mathematics and Physics, University of Campania “Luigi Vanvitelli”, 81100 Caserta, Italy; bahar.faramarzi@unicampania.it; 3 Dipartimento di Fisica “E. Pancini”, Università Federico II di Napoli, 80126 Napoli, Italy; lorenzo.manti@unina.it; 4 Istituto Nazionale di Fisica Nucleare (INFN), Sezione di Napoli, 80126 Napoli, Italy

**Keywords:** sphingolipidomics, extraction methods, mass spectrometry, HepG2 cells, X-ray

## Abstract

An analytical method based on tandem mass spectrometry-shotgun is presently proposed to obtain sphingolipidomic profiles useful for the characterization of lipid extract from X-ray-exposed and unexposed hepatocellular carcinoma cells (HepG2). To obtain a targeted lipidic profile from a specific biological system, the best extraction method must be identified before instrumental analysis. Accordingly, four different classic lipid extraction protocols were compared in terms of efficiency, specificity, and reproducibility. The performance of each procedure was evaluated using the Fourier-transform infrared spectroscopic technique; subsequently, the quality of extracts was estimated using electrospray ionization tandem mass spectrometry. The selected procedure based on chloroform/methanol/water was successfully used in mass spectrometry-based shotgun sphingolipidomics, allowing for evaluation of the response of cells to X-ray irradiation, the most common anticancer therapy. Using a relative quantitative approach, the changes in the sphingolipid profiles of irradiated cell extracts were demonstrated, confirming that lipidomic technologies are also useful tools for studying the key sphingolipid role in regulating cancer growth during radiotherapy.

## 1. Introduction

In recent years, the interests of researchers have been focused on sphingolipids (SPLs), a highly diversified and complex class of lipids. In addition to their typically structural function (they are a small but vital fraction of membrane lipids), this class of lipid molecules plays a wide variety of cellular and systemic bioactivities, including cell signaling, transport, protein trafficking, growth, differentiation, and apoptosis [1]. Thus, several studies have highlighted the potentially critical role of SPL on the pathogenesis of various diseases (e.g., Alzheimer’s disease [2,3,4], cancer [5,6], metabolic syndrome [7,8]).

In the complexity of the cellular metabolic system, SPLs are continuously interconverted. The SPL metabolism consists of a highly complex network (see Figure S1 in the Supplementary Materials), in which the metabolic hub is ceramide (Cer), occupying a central position in the biosynthesis and catabolism of other bioactive SPLs [9,10,11,12,13]. Different pathways converge in the formation of Cer: de novo biosynthesis, from initial condensation of the palmitoyl-CoA and the amino acid serine; the salvage pathways, from the recycling of sphingosine (So) and complex sphingolipids; and the hydrolysis of membrane sphingomyelin (SM). Once produced, the Cer can accumulate or be converted under the action of different enzymes. Cer can be reversed in SM by sphingomyelin synthase or glycosylated to glycosphingolipids (GlcSph). In addition, the deacylation of Cer forms So, an important regulatory molecule of several processes, which can be re-converted to Cer or phosphorylated by sphingosine-kinase to sphingosine 1-phosphate (S1P). The latter can be degraded through two different reactions: the reversible dephosphorylation to So, catalyzed by S1P phosphatase, or the irreversible degradation in phosphoethanolamine and hexadecenal (palmitaldehyde). These molecules can be reused for the biosynthesis of phosphatidylethanolamine, while hexadecenal can also be oxidized to palmitate, thus re-entering the lipid metabolism. In conclusion, in the complex network of the SPL metabolism, the hydrolysis of S1P into the non-sphingolipid molecules mediated by S1P lyase is the only irreversible reaction that does not render SPL metabolic intermediates [5,10,11,12,13,14].

Since many of the metabolic pathway intermediates described above represent bioactive molecules involved in the modulation of several important biological processes, the cells must finely regulate the intracellular levels of these molecules. This is achieved by regulating the activity of the enzymes involved in their biosynthesis and degradation in response to extracellular stimuli. As a classic example, oxidative stress induces So and/or Cer synthesis via de novo and SM hydrolysis, resulting in increased levels of So and Cer and induction of apoptosis by these molecules [15]. Conversely, many tumors show increased Cer metabolism mainly due to increased activities of glucosylceramide synthase (GlcCerS), sphingomyelin synthase (SMS), ceramide kinase (CerK), and/or sphingosine kinase (SphK), which increases the synthesis of sphingolipids with pro-survival properties [5,16,17]. In particular, the SphK1 isozyme, with its isoforms SphK1-a and SphK1-b, is over-expressed in numerous types of cancer cells, leading to an increase in S1P levels and a concomitant reduction in Cer levels, thus suppressing the pro-apoptotic signals and simultaneously creating a survival signal [18,19]. The dynamic balance between these two SPL is the so-called “sphingolipid rheostat” [20], where cell fate depends not on the absolute levels but on the relative amounts of these antagonistic metabolites. Consequently, several studies have been performed targeting the enzymes that regulate SPL levels and developing new therapeutic strategies to maintain the correct dynamic balance among SPL.

The specific application of lipidomics on SPL, called “sphingolipidomics”, allows for the complete identification of SPLs present in a tissue, organelle, or cell, and the evaluation of any alterations (abnormalities in metabolism) in response to physiological and pathological conditions [21]. Thus, the study of the sphingolipidome can provide vital information on cellular homeostasis, as well as the pathogenesis of diseases and cancer [1,22,23]. Sphingolipidomics represents a valid tool for identifying and monitoring these new diagnostic and/or prognostic biomarkers [24].

Important advances in mass spectrometry and modern software for lipid identification have led to increased sensitivity and mass accuracy, making it possible to rapidly detect and subsequently identify many lipid species. However, to obtain a targeted lipidic profile from a biological system, the extraction method must be optimized before instrumental analysis. For this reason, no single protocol has been established. The choice of the proper extraction procedure depends on the specific biological matrix, the purpose of analysis, and the experimental design [25]. The most used technique of lipid isolation from biological samples is lipid extraction with organic solvents in which hydrocarbon chains have high solubility [26]. The most common methods involve the addition of chloroform and methanol to an aqueous biological solution or freeze-dried tissue [27,28]. Modifications have been made to the chloroform–methanol extraction procedure to maximize the efficient extraction of lipids of specific interest and to utilize inexpensive and less toxic solvents. Löfgren et al. (2012) [29] proposed the use of butanol/methanol, a method later improved by Cruz et al. (2016) [30]; meanwhile, many investigators developed faster single-step procedures based on a mixture of isopropanol, hexane, or ethyl acetate [31,32,33]. At last, Shaner et al. (2009) [34] investigated an analytical protocol characterized by repeated re-extraction of the samples to recover the specific lipids.

In the present paper, we propose an analytical method based on tandem mass spectrometry-shotgun to obtain sphingolipidomic profiles useful for the characterization of lipid extracts from X-ray-exposed and unexposed hepatocellular carcinoma cell lines (HepG2). For this reason, we compared four lipid extraction protocols for HepG2. The different solvent systems were tested in terms of efficiency, specificity, and reproducibility. A preliminary assessment of these characteristics was performed by adopting Fourier-transform infrared (FT-IR) spectroscopy following the literature indications [35,36,37]. Subsequently, the identification and profiling of SPLs were carried out using electrospray ionization tandem mass spectrometry-shotgun (ESI-MS/MS), the leading technology for providing structural information on multiple biomolecules simultaneously [38,39,40,41,42,43,44,45]. ESI-MS/MS can be used to directly characterize crude lipid extracts without prior chromatographic purification reaching high accuracy as demonstrated in several papers [40,41,42,43,44,45]. Tandem mass spectrometry analysis of the fragment pattern of the sphingolipid molecular species provides valuable information on the molecular structures, enabling their identification. On the other hand, chromatographic separation of lipid extracts reduces the complexity of mass spectra and requires more demanding and sophisticated quantitation strategies than direct infusion MS/MS [46].

The selected analytical procedure was applied on HepG2 cells to investigate whether a sphingolipidomic approach is useful to characterize extracts of cells irradiated with X-ray as photons that are commonly used in anticancer radiotherapy. As reported in the literature [47,48,49], ionizing radiation should induce a rapid increase in Cer levels and a concomitant decrease in the pool of SM in the plasma membrane, independently from the DNA damage. Also, an increase in So levels can be rapidly observed after exposure to X-ray [49,50].

Using a relative quantification approach, changes in the sphingolipidomic profiles of the extracts of irradiated cells are demonstrated, and the identified analytical method qualifies a valid analytical tool to study sphingolipid-related processes.

## 2. Results and Discussion

### 2.1. Optimization of the Best Extraction Procedure of Sphingolipids from Cellular Pellets

The lipid extracts obtained from cell pellets according to the four procedures reported in Section 3.2.3 were analyzed by FT-IR and electrospray ion source mass spectrometry (ESI-MS/MS).

#### 2.1.1. Evaluation of Lipid Extraction Efficiency by FT-IR Spectroscopy

Aliquots of lipid extracts were analyzed by FT-IR spectroscopy. This technique allowed for a preliminary quantitative comparison of the lipid extraction efficiency of the different methods. Due to their complex chemical composition, lipids absorb in many different regions of the IR spectrum. The presence of characteristic lipid bands, such as aliphatic group stretch (3100–2800 cm^−1^), C = O ester stretch (about 1740 cm^−1^), or phosphate PO_2_^-^ stretch (about 1235 cm^−1^), allows for the qualitative and quantitative analysis of lipid content [51].

**Figure 1:**
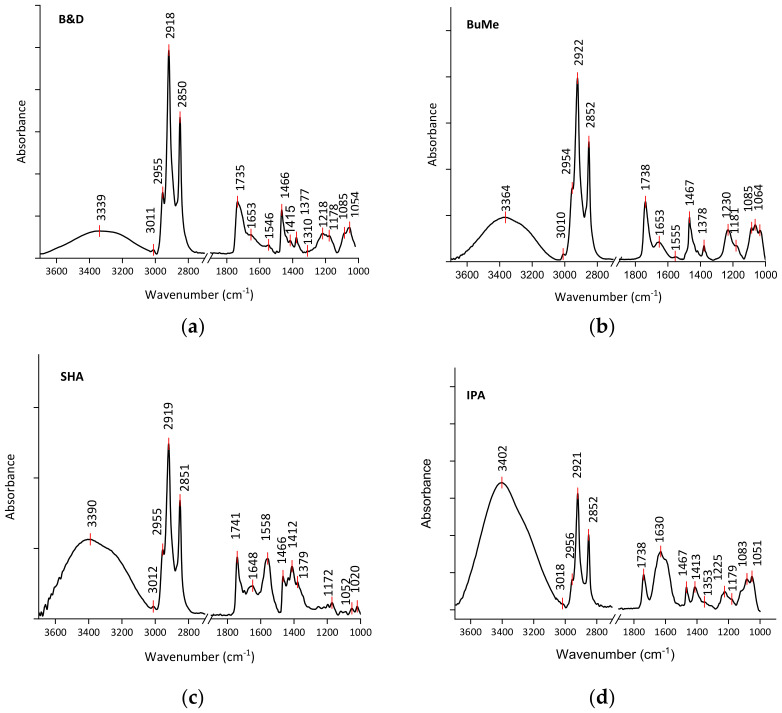
Comparison of average FT-IR spectra for samples obtained from unirradiated HepG2 cells by different lipid extraction methods: (**a**) B&D; (**b**) modified BuMe; (**c**) SHA; (**d**) IPA.

In Figure 1, the average of normalized FT-IR spectra of lipid samples extracted from HepG2 cells using the above-mentioned procedures are reported. As is evident, it is possible to notice the contributions due to the above-mentioned functional groups linked to the lipids. In Table S1, the assignments of the main peaks present in the FT-IR spectra are reported [52,53,54].

**Figure 2:**
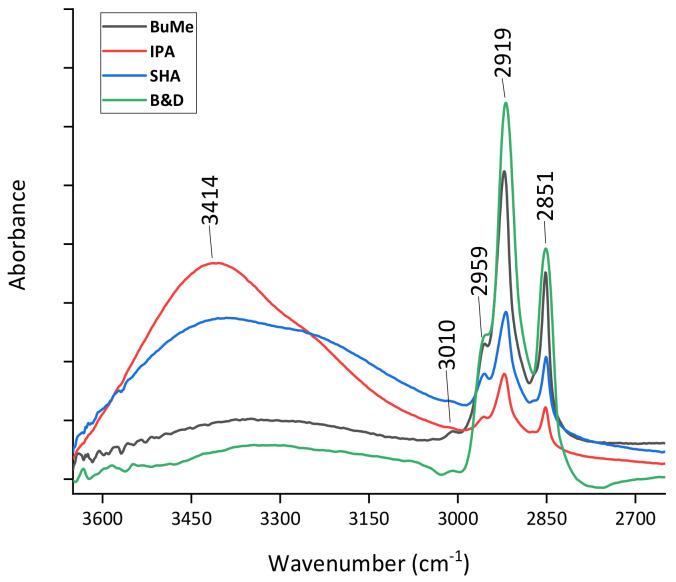
High-wavenumber region of average spectra acquired from lipid samples extracted using the four adopted procedures.

To carry out a more accurate analysis and to highlight the possible variations between the different extraction methods, it can be useful to focus on the high-wavenumber region of the spectra reported in Figure 2 where it is possible to note the presence of the contributions located around 2959, 2919, and 2851 cm^−1^ that can be related to the CH_3_ asymmetric stretching, CH_2_ asymmetric stretching, and CH_2_ symmetric stretching modes, respectively.

Different criteria can be adopted to evaluate the efficiency of the different lipid extraction methods. For example, the intensity of the peak located at around 2919 cm^−1^ can be assumed to be an indicator of extraction efficiency. Using this criterium, it is evident that the B&D method can be considered the most efficient. This result is confirmed by evaluating the intensity of the 2870–2850 cm^−1^ band as suggested in the literature [36,55,56]. In addition, around 3400 cm^−1^, a contribution essentially due to N-H stretching mode likely ascribed to the residual presence of some protein component (Amide A) is more largely present only in the spectra of the samples extracted using SHA and IPA methods. This indicated that the B&D and modified BuMe methods are also characterized by the higher purity of the extracts.

#### 2.1.2. Characterization of Lipid Extracts from HepG2 Pellets Employing Different Protocols Using Tandem Mass Spectrometry-Shotgun

**Figure 3:**
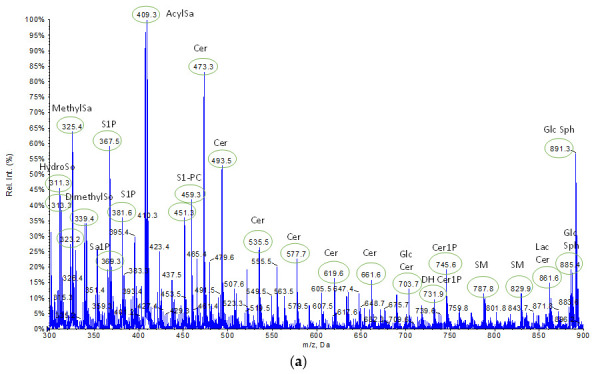

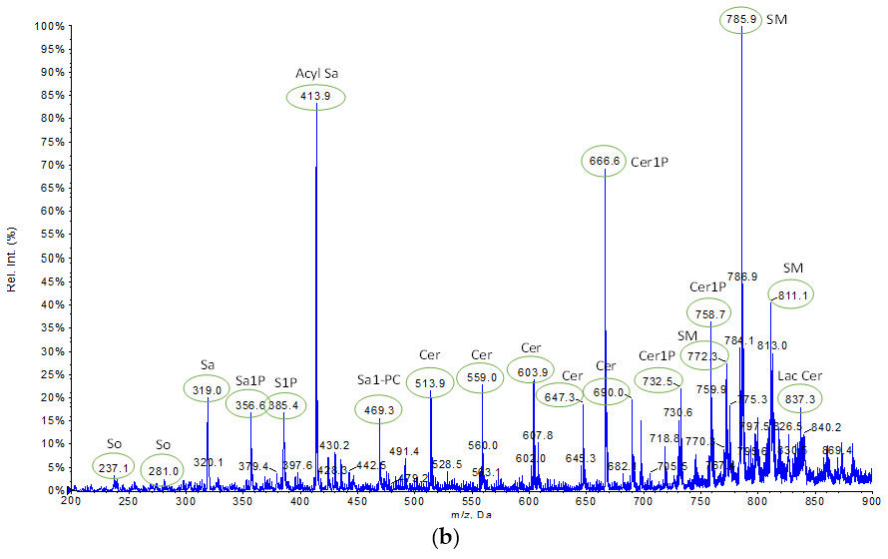
Representative mass spectra of samples obtained by B&D method using unirradiated HepG2 cells: (**a**) in negative-ion mode; (**b**) in positive-ion mode.

Each lipid extract from cell pellets was analyzed using tandem mass spectrometry to identify the molecular lipid species and evaluate the relative extractability. The analyses were carried out by direct infusion of each sample in negative- and positive-ion mode to acquire full mass spectra of the SPL extracts. Three different samples were analyzed for each experimental group. All molecular ions were subject to fragmentation by product ion MS/MS. Using specific database (LipidMaps Database, http://www.lipidmaps.org accessed on 26 July 2023), MS/MS interpretation for each ion yielded the identification of the SPL subclasses: several sphingoid bases, such as sphingosine (So) and sphinganine (Sa), dihydroxy sphingosine and related phosphorylate forms, such as sphinganine-1-phosphate (Sa1P) and sphingosine-1-phosphate (S1P), ceramides (Cer) and ceramide 1-phosphate (Cer1P), sphingomyelins (SM), and glycosphingolipids (GlcSph). In Table 1 and Table 2, we summarize the *m*/*z* of molecular ions and the relative product ions that specifically identified the SPL species, while Figure 3 shows the representative spectra obtained operating in negative-ion mode (a) and positive-ion mode (b). SPL profiles of Figure 3 refer to the extract obtained by the B&D method, while the mass spectra of the extracts obtained by the other methods are reported in Figures S2–S4 of the Supplementary Materials.

In the class of sphingolipids, the defining component is a sphingoid base skeleton to which an amide-linked fatty acid and (or) a primary hydroxyl head group may be attached. The backbone can vary in length/branching of the alkyl chain, number, and position of double bonds, number, and position of hydroxyl groups, etc. Consequently, like S1P or Cer, they are not molecules with a single exact mass, but instead, each one represents a multiplicity of structures differing in the number of C and/or type of amino-linked fatty acids. Therefore, while identifying the various classes of SPL, often it was not possible to assign a single exact mass.

The sphingoid bases are predominantly present in spectra obtained operating in negative-ion mode in a mass range of *m*/*z* 311.3–339.4, as well as the relative phosphorylated forms, identifiable by the characteristic fragments at *m*/*z* 78.8 (loss of the phosphate group). Cer, on the other hand, was detected in both negative- and positive-ion modes and presented the characteristic fragment at *m*/*z* 264.4 in product ion mode. In particular, the peaks assigned to Cer have a higher relative intensity in positive-ion mode where the most abundant Cer possessed C18 sphingoid base backbones with an N-acyl chain length ranging from 14 to 26 carbons, e.g., Cer(d18:1/14:0) at *m*/*z* 513.9, Cer(d18:1/17:0) at *m*/*z* 559.0, Cer(d18:1/21:3) at *m*/*z* 603.9, Cer(d18:1/24:1) at *m*/*z* 647.3, and Cer(d18:1/26:1) at *m*/*z* 690.1. Of note, a rare Cer with a minor sphingoid backbone, Cer(d14:1/15:0), presented higher intensity than other Cer in spectra obtained in negative-ion mode. Also, a total of four Cer1P molecules were identified, all with C18 sphingoid backbones. Specifically, Cer1P(d18:1/19:0) at *m*/*z* 666.6 had significative intensity.

According to the literature [56], SM are the most abundant class of SPLs in HepG2 cells. SM were detected in negative-ion mode at *m*/*z* 787.8 end at *m*/*z* 829.9, while in positive-ion mode at *m*/*z* 772.3, at *m*/*z* 758.7, at *m*/*z* 785.9, end at *m*/*z* 811.1, showing the identifying fragment ions at *m*/*z* 184.4 due to loss of the phosphorylcholine headgroup. Mainly, SM possessed a C18 sphingoid base backbone; d18:1 is the major type and SM (d18:1/22:0) at *m*/*z* 785.9 is the most abundant species. Also, the peak assigned to Acylsphinganine (AcylSa, dihydroceramide), a precursor of ceramide in de novo biosynthesis, present both in negative-ion mode at *m*/*z* 409.3 and in positive-ion mode at *m*/*z* 413.9, is of significant intensity.

#### 2.1.3. Comparison of Extraction Procedures from HepG2 Pellets Using Tandem Mass Spectrometry-Shotgun

The lipid extraction protocols were characterized, and their performances were compared evaluating efficiency and specificity, recovery, precision, and reproducibility.

##### Efficiency and Specificity

**Figure 4:**
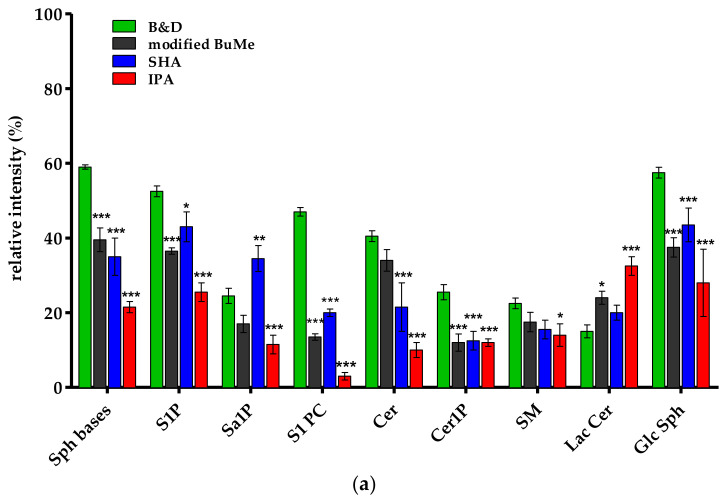

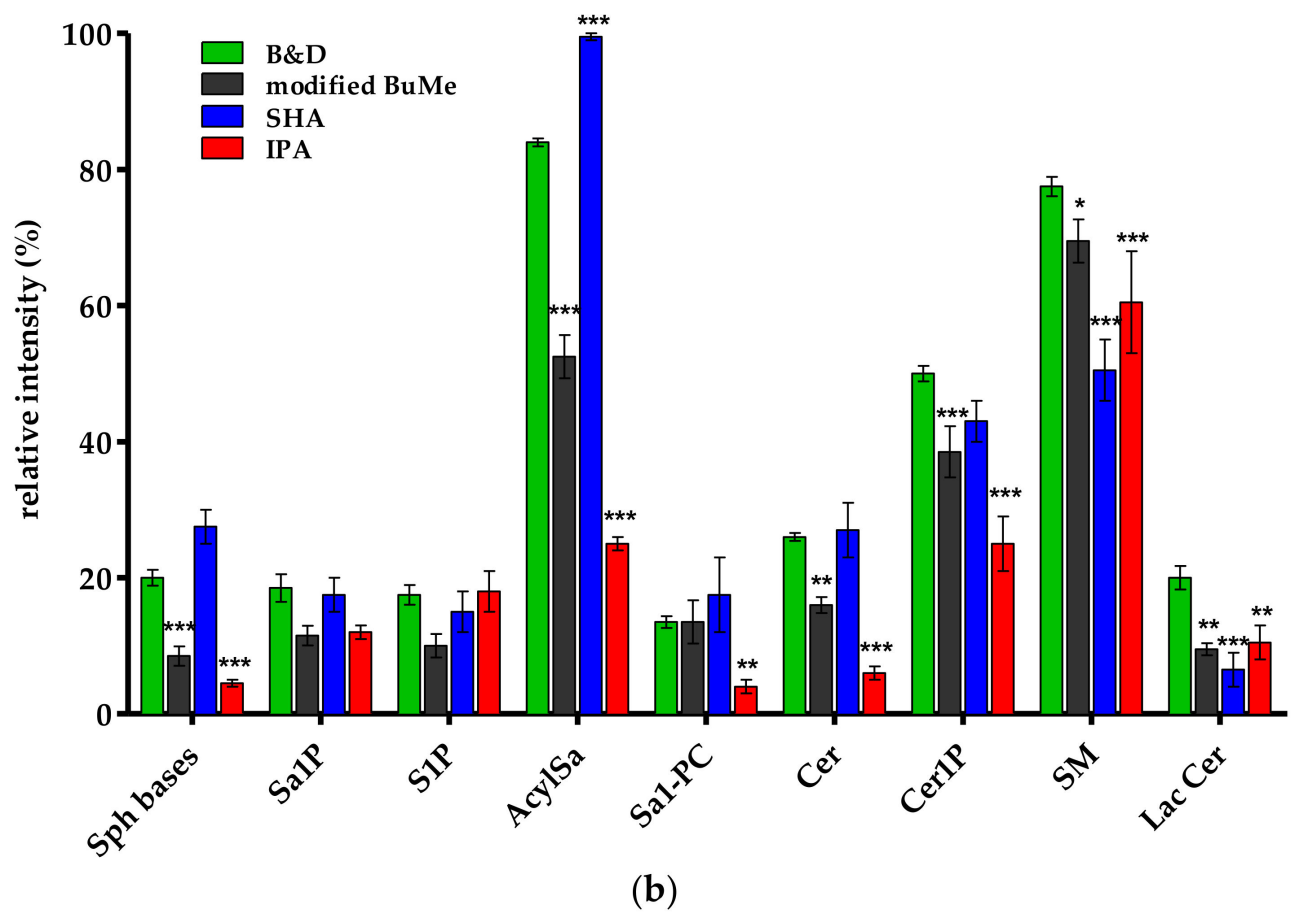
Comparison of relative intensity calculated from mass spectra of samples obtained by different lipid extraction methods using unirradiated HepG2 cells: (**a**) in negative-ion mode; (**b**) in positive-ion mode. Error bars represent SD; the asterisks indicate that a significant difference relative to B&D method occurred at *** *p* < 0.001; ** *p* < 0.01; * *p* < 0.05.

In Figure 4, the relative intensities of the peaks assigned to the various sphingolipid subclasses are reported. Data obtained from the analysis of three different samples for each experimental group are expressed as the mean ± standard deviation (SD). Statistically significant differences were assessed by one-way ANOVA followed by an unpaired, two-tailed independent sample t-test. A *p*-value less than 0.05 was considered statistically significant. Statistical analysis was performed using GraphPad Prism 5.0 H statistical software (GraphPad Software Inc., La Jolla, CA, USA).

Figure 4a refers to the negative-ion full spectra. The relative percentages were calculated with respect to the peak of maximum intensity, i.e., that relating to AcylSa (at *m*/*z* 409.3), a metabolite in the biosynthesis of ceramides de novo. Figure 4b shows the relative intensities calculated from the positive-ion full spectra. For the SHA method, the relative percentages have been calculated concerning the peak assigned to AcylSa (at *m*/*z* 413.9), while for B&D, modified BuMe, and IPA methods, the relative percentages were calculated to the SM peak, which had the maximum intensity at *m*/*z* 785.9. In this case, the graph shows the relative intensities of the peaks assigned to the SM class, calculated as the ratio between the mean intensity of the various peaks assigned to this class and the intensity of the single maximum peak at *m*/*z* 785.9.

Firstly, the comparison of the data shown in Figure 4a,b confirms that for all extraction methods, the sphingoid bases and the related phosphorylated forms (S1P and Sa1P) mainly ionize in a negative-ion mode, as do the Lac Cer and GlcSph, while Cer1P and SM ionize in a positive mode [44,56]. The relative intensities of Cer are apparently higher in negative-ion spectra. This is due to the presence of the rare Cer(d14:1/15:0), which has a higher intensity at m/z 473.3 in negative-ion spectra. In fact, the Cer (d18:1) subclass, as shown in Figure 3, ionizes best in the positive-ion mode [57,58].

One-way ANOVA was used to test whether the relative intensity of SPL classes using the B&D method were significantly different from the modified BuMe, SHA, or IPA methods. In negative-ion mode, there were significant differences in relative intensity between extraction methods for Sph bases, S1P, S1PC, Cer1P, and GlcSph species (*p* < 0.05). The relative intensity of LacCer was significatively higher in the modified BuMe and IPA methods than the B&D method (*p* < 0.05 and *p* < 0.001, respectively), while the latter was more efficient in Cer and SM extraction but yielded slightly higher intensity compared to the modified BuMe method, which did not reach significance (*p* >0.05).

In positive-ion mode, there were no significant differences in relative intensity between the extraction methods for Sa1P, S1P, and Sa1-PC species (*p* > 0.05). Sph bases, AcylSa, Cer, Cer1P, SM, and LacCer were significantly higher in the B&D method compared to the modified BuMe and IPA methods. Instead, the SHA method produced similar, if not greater, yields than the B&D method.

Overall, both datasets highlight that the B&D method was more effective in terms of the extraction of more polar lipids as an SPL class. The main reason for this is likely beyond the larger solvent volume, the use of a chloroform/methanol mixture in the first step of the extraction procedure. The presence of methanol in a solvent solution increases the relative polarity of the organic phase, disrupting the hydrogen-bonding networks or electrostatic forces between lipids and proteins, then effectively solubilizing the polar lipids [25]. Nonpolar chloroform mediates the diffusion through the cell wall, dissolves the neutral lipids and its water-immiscible properties help in the formation of a biphasic system. The SHA method produced similar and sometimes greater yields than the B&D method, but by providing a double extraction in parallel, it is laborious and expensive in terms of time and solvent consumption. Consequently, the modified BuMe method is preferrable to the latter, even though it has slightly lower yields. The butanol can replace the more toxic chloroform, but it is less efficient and slower to evaporate. Finally, the IPA method is certainly shorter and less complex to process, but it is also less applicable to obtaining a good lipidomic profile. Isopropanol may be weaker in disrupting such membrane-lipids-protein than methanol due to its larger hydrophobic property, affecting the extraction efficiency of polar lipids as SPLs.

##### Recovery

To assess recoveries for each method, additional experiments were performed using cell samples spiked before and after extraction with a standard solution of S1P, Cer, and SM. The SPL extracts were directly analyzed by tandem mass spectrometry-shotgun in precursor ion scanning mode by selecting product ion at *m*/*z* 78.8, at *m*/*z* 264.4, and at *m*/*z* 184.4 for S1P, Cer, and SM, respectively. By comparing the ratio of peak areas of standards spiked prior to and after extraction, the average recoveries were 79, 69, 73, and 63% for the B&D, modified BuMe, SHA, and IPA methods, respectively. The highest recovery for the B&D method was achieved with the S1P (>80%) and the lowest for the less polar Cer (<75%).

##### Precision and Reproducibility

Lipidomics studies based on mass spectrometry-shotgun analyses are characterized by comparative approach, i.e., the comparison of SPL profiles between control and treated samples. Consequently, the precision and reproducibility of the extraction protocols are extremely important and must be investigated.

The intra-assay precision, as assessed by triplicate analytical runs of the same sample, was found within a SD% range of 1.4–5.0% for B&D method, 2.1–9.2% for modified BuMe, 1.4–9.1% for SHA method, and 1.5–12.7% for IPA method. All the results were satisfactory, but the B&D method is highly reliable.

**Figure 5:**
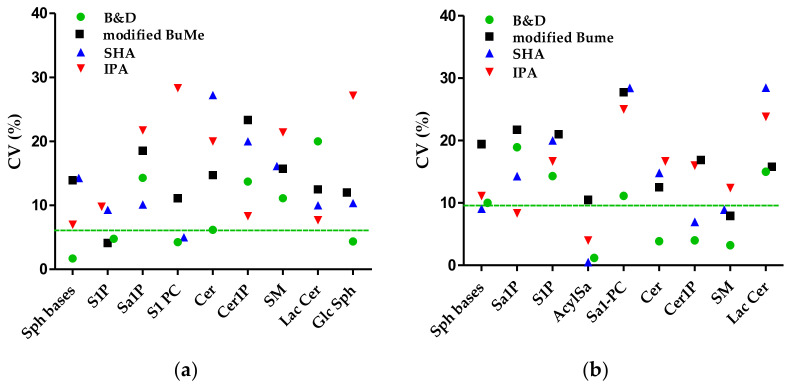
Inter-assay reproducibility, expressed as percentage coefficient of variation CV%, of extraction methods for all SPL subclass in negative-ion (**a**) and positive-ion (**b**) mode. The dotted line represents the median CV% of the B&D method.

By comparing the analyses of the three replicates of HepG2 cells, the inter-assay reproducibility of extraction methods for all SPL subclass in negative- and positive-ion mode was calculated as CV% (Figure 5).

All values were found to be less than 30%, consistent with the other reports [59,60]. In particular, the inter-assay reproducibility of B&D method was very good, with the median CV% across all SPL subclasses below 10.0% (6.2% and 9.8% for negative-ion mode and positive-ion mode, respectively), and lower than that obtained for the other methods. The median inter-assay CV% for SPL in negative-ion mode were 13.9, 10.34, and 19.7% for the modified BuMe, SHA, and IPA methods, respectively; while in positive-ion mode they were 16.8, 14.3, and 15.8%, respectively. When analyzing the values of target SPL classes (i.e., S1P, Cer, Cer1P, and SM), the B&D and modified BuMe methods were the most reproducible for S1P detected in negative-ion mode (CV < 5%). For Cer, Cer1P, and SM detected in positive-ion mode, the CV% values of the B&D method were still < 5%, while the IPA proved to be the worst method with values between 12 and 17%.

Therefore, it can be concluded that the best analytical method to obtain a consistent and accurate sphingolipidomics profile from the cellular model used in this study, specific for identifying the main classes of SPLs, is the classic extraction method chloroform/methanol/water of Bligh and Dyer [28] associated with ESI tandem mass spectrometry-shotgun.

### 2.2. Analyses of Lipidomic Profiles of Irradiated HepG2 Cells

**Figure 6:**
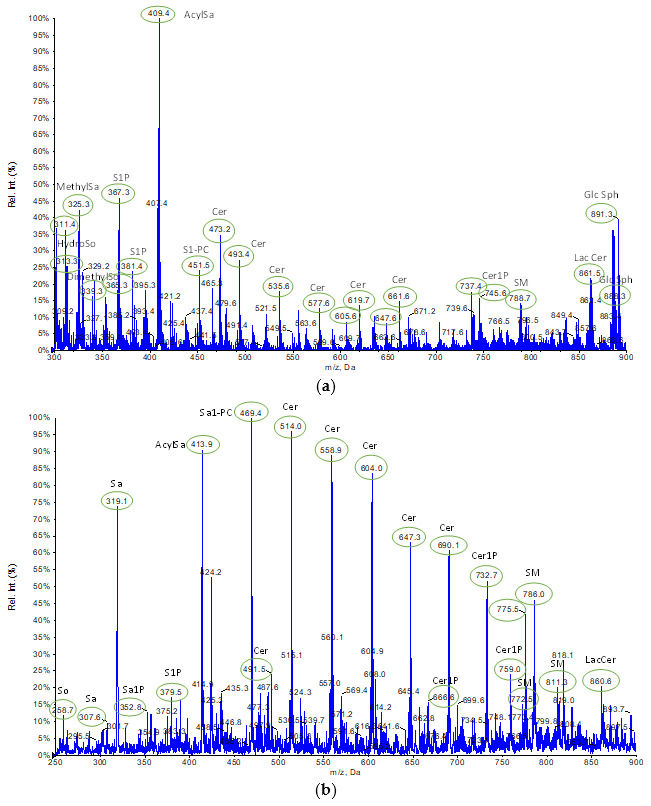
Representative mass spectra of samples obtained by B&D method using irradiated HepG2 cells: (**a**) in negative-ion mode; (**b**) in positive-ion mode.

The optimized analytical method was applied to evaluate the changes in the SPL profile obtained from extracts of cells subjected to X-ray irradiation treatment at 2 Gy compared to the ones of unexposed cells. The extraction procedure was performed according to the B&D method. All the spectra show the presence of the peaks assigned to the previously identified molecules but with different intensities. In Figure 6, the representative SPL profiles of extract from irradiated cells are represented.

By comparing the spectra obtained in negative-ion mode (Figure 3a vs. Figure 6a), a significant decrease in the intensity of the peaks assigned to the sphingoid bases and their phosphorylated forms, in particular S1P and Sa1P, is evident in the SPL profile relative to the irradiated cell. Also, the peaks assigned to the Cer show an average reduction in intensity, although only the peaks at *m*/*z* 473.2 and *m*/*z* 493.4 are significantly less intense. Finally, a slight decrease in the intensity of the peaks relating to Cer1P and GlcSph is noted in irradiated cell profile, while those relating to SM maintain an almost constant intensity. By relating these results to the respective pro-proliferative roles of the sphingolipids S1P, Cer1P, SM, and GlcSph, effective antitumor action of the 2-Gy therapeutically relevant radiation dose on the HepG2 cell system can be highlighted, even if not supported by a clear increase in the intensity of the peaks related to the Cer, whose role has been widely recognized as pro-apoptotic. However, this is evident by comparing the spectra obtained in positive-ion mode (Figure 3b vs. Figure 6b), which represents the best operating mode to ionize Cer, in agreement with the literature [56,57]. Indeed, in the spectra of the irradiated cell extracts, a clear increase in intensity in the mass range of the Cer (*m*/*z* 514.0, *m*/*z* 558.9, *m*/*z* 604.0, *m*/*z* 647.3, *m*/*z* 690.1) is recorded. Equally interesting is the increase in the intensity of the peaks assigned to Sa (*m*/*z* 319.1) and AcylSa (*m*/*z* 413.9), indicating the progressive biosynthesis of Cer via de novo. On the other hand, the average intensity of the Cer1P peaks (*m*/*z* 666.6, *m*/*z* 758.9) and the SM peaks (*m*/*z* 785.9, *m*/*z* 811.1) decreased. Thus, the significant reduction in peak intensity at *m*/*z* 786.1 highlights a shift in the balance in favor of the Cer subclass over the SM, presumably due to the activation of SM catabolism. The ionizing radiation can induce cell damage through two independent signaling pathways, leading to an increase in intracellular Cer [47,48,49,61]. A rapid and transitory generation of Cer from the acid SMase activation is observed within minutes following radiation exposure and is independent of the DNA damage. Several hours after irradiation, a second wave of Cer accumulation is observed as a consequence of DNA damage-dependent Cer synthase activation. In the present study, after irradiation, the cells were immediately removed from the flasks and pelleted by centrifugation. Therefore, our preliminary results emphasize the early effects of ionizing radiation and suggest that the enhancement of ceramide-mediated apoptotic processes generated by alterations in the cellular membranes occurred in the irradiated cells [62]. This apoptotic signaling represents an alternative to nuclear signaling generated by direct DNA radiation damage.

In support of this thesis, we refer to the “sphingolipid rheostat” model, for which the fate of the cell is determined not by the absolute levels but by the relative quantities of the antagonistic metabolites S1P and Cer [20]. Therefore, the ratios between the total area of the peaks assigned to S1P with respect to the total area of ceramide peaks (S1P/Cer) were calculated. The values show a balance in favor of the Cer for the irradiated cells compared to the control cells (0.46 vs. 1.85, respectively) and further confirm that Cer, once generated, leads to the activation of a cell-death signaling cascade, and the conversion of Cer to S1P does not reverse this process. 

In conclusion, using the optimized analytical methodology, it was possible to highlight the alterations of the SPL profile of HepG2 cells submitted to physical therapy with radiation. The method results in a valuable analytical tool to use in (sphingo)lipidomic studies. In addition, the results from the irradiated samples warrant further radiobiological studies to explore the potential of lipidomics also in novel radiotherapy regimes [63]. Accurate determination of the levels of Cer and S1P in the tumor microenvironment will help design therapies targeting ceramide and S1P signaling for the treatment of cancer patients.

## 3. Materials and Methods

### 3.1. Materials

Human hepatocellular carcinoma cells (HepG2), Dulbecco’s Modified Eagle Medium (DMEM), fetal bovine serum (FBS), penicillin, and L-glutamine were purchased from Merck (Darmstadt, Germany) and used without any further treatments.

Heptane (Hept), isopropanol (IPA), ethyl acetate (EtAc), butanol (Bu), and all reagents were procured from Merck (KGaA, Darmstadt, Germany). MS grade reagents, including ultrapure water, methanol (MeOH), and chloroform (CHCl_3_) were acquired from Romil (ROMIL Ltd., Cambridge, UK). S1P (d18:1), Cer (d18:1, 18:0) and SM (d18:1, 20:0), Avanti Polar Lipids standards, were purchased from Merck (Darmstadt, Germany).

### 3.2. Methods

#### 3.2.1. Cell Culture Preparation

The HepG2 cells were cultured in DMEM, supplemented with 10% heat-inactivated FBS, 100 U/mL penicillin, 100 µg/mL streptomycin, and 1% L-glutamine. The cells were grown in a humidified atmosphere of 95% air/5% CO_2_ at 37 °C in standard tissue culture flasks. Cells were routinely subcultured to obtain cellular samples derived from one single batch and to avoid inter-batch variation.

To prepare cell samples for sphingolipid extraction, the cells were detached by prewarmed trypsin, rinsed three times in PBS, and pelleted by centrifugation 1200 rpm for 5 min. Some samples were subjected to irradiation according to the procedure reported below, while other ones were used for extraction procedure characterization. For each experimental groups, three replicates of HepG2 cells were prepared. All pellets were stored at −20 °C until lipid extraction. 

#### 3.2.2. X-ray Irradiation Treatment

X-ray irradiations were performed at the Radiation Biophysics laboratory, Physics Department, University of Naples Federico II using a STABILIPAN 2 machine (Siemens, Munich, Germany), where photons were produced by a Thomson tube (TR300F, 250kVp) and filtered by a 1 mm thick Cu foil at a dose rate of about 1.3 Gy/min [64]. Routine dosimetry was performed to ensure dose uniformity with less than 5% variation within the radiation field [65]. A 2 Gy dose was chosen for the present investigation as representative of a typical fractionated radiotherapy treatment. After irradiation, cells were removed from the flasks by trypsinization and pelleted by centrifugation as reported above.

#### 3.2.3. Lipid Extraction Protocols

Prior to the extraction procedure, all pellets were sonicated with 1 mL of 0.9% saline buffer 2 × 20 Hz for 20 s.

##### B&D Method

Following the original protocol [28], SPLs were extracted by the addition of 3.75 mL of CHCl_3_:MeOH (1:2, *v*/*v*) to the cell sample with occasional vortex mixing for 10 min. Then, 1.25 mL of CHCl_3_ was added, and after 5 min of vortex mixing, phase separation was achieved by adding 1.25 mL water. The sample was centrifuged for 5 min at 1000 rpm; the upper (aqueous) phase was removed, while the organic phase was dried under stream of nitrogen and resuspended in 1 mL of MeOH until MS analysis.

##### BuMe Modified Method

As reported by Cruz et al. (2016) [30], 300 μL of Bu/MeOH solution (3:1, *v*/*v*) was added to a test tube containing the sample and vortexed for 1 min. Then, 150 μL of Hept/EtAc (3:1, *v*/*v*) was added and, after 1 min of vortex mixing, another 150 μL of Hept/EtAc was added. Phase separation was achieved by adding 300 μL of LiCl solution (50 mM). After 10 min of centrifugation at 2700 rpm, the upper organic phase was collected while the aqueous phase was reextracted twice, first with 320 μL of Hept/EtAc (3:1), then with 250 μL of the same solvent. After each addition of Hept/EtAc, the solution mixture was vortexed and centrifuged at 2700 rpm for 10 min, and the resulting upper organic layers were collected and pooled with the previous organic phases. The extracts were dried under stream of nitrogen and resuspended in 1 mL of MeOH until MS analysis.

##### SHA Method

This procedure was based on Shaner et al. (2009) [34]. After adding 0.5 mL of MeOH and 0.25 mL of CHCl_3_, the cell sample was vortexed for 10 min end then incubated at 48 °C overnight. After cooling, 75 µL of KOH in MeOH (1 M) was added and incubated in a stirred water bath for 2 h at 37 °C. An aliquot (0.4 mL) was placed in a test tube, and after centrifugation, the supernatant was collected while the residue was again extracted with 1 mL of MeOH:CHCl_3_ (1:2, *v*:*v*) and then centrifuged. In parallel, 1 mL of CHCl_3_ and 2 mL of H_2_O were added to the remaining original extract, and after vortexing and centrifugation, the lower organic layer was collected in a test tube. The upper phase was extracted with 1 mL of CHCl_3_ and pooled to the previous organic phase. Finally, the supernatants were combined, the solvents were removed, and the dried residue was reconstituted in 1 mL of MeOH.

##### IPA Method

This method was an adaptation of the monophasic procedure described in Hammad et al. (2010) [32]. A total of 2 mL of IPA/EtAc/H_2_O (60:10:30) was added to the cell pellet and vortexed for 1 min. The sample was sonicated twice (20 Hz for 1 min) and centrifugated at 4000 rpm for 10 min at 24 °C. The upper organic phase was collected, dried under stream of nitrogen, and resuspended in 1 mL of MeOH until MS analysis.

#### 3.2.4. FT-IR Spectral Analysis

The cellular extracts were resuspended in methanol, and drops of a few microliters were placed on CaF_2_ windows and left to dry before spectra acquisition. FTIR spectra were obtained in transmission mode using a Perkin Elmer Spectrum One spectrometer equipped with a Perkin Elmer Multiscope system infrared microscope and a liquid nitrogen MCT (mercury–cadmium–telluride) detector. All spectra were collected using 64 scans in the range of 4000 to 1000 cm^−1^ with a 4 cm^−1^ spectral resolution. The acquired spectra were baseline-corrected and normalized using the standard normal variate (SNV) method [66].

#### 3.2.5. Mass Spectrometry Analysis

Sample analysis was performed on a triple quadrupole spectrometer (Sciex, Darmstadt, Germany) equipped with a TurboIon electrospray source operating in positive- and negative- ion modes. Lipid extract samples were directly infused into the ESI source using a syringe pump at a flow rate of 10 μL/min. The acquisition parameters were set as follows: ion-spray (IS) voltages: 5.5 kV and −4.5 kV, respectively for positive- and negative-ion modes; curtain gas (CUR): 20 psi; nebulizer gas (GS1) and dry gas (GS2): 20 psi; and interface heater temperature (TEM): 100 °C. Other parameters were set at 60 V for declustering potential (DP), 10 V for entrance potential (EP), while collision energy (CE) was set an optimized value for each molecular species, as reported in Table 1 and Table 2. To obtain a full spectrum, MS scanning was acquired from *m*/*z* 200–900 with an accumulation time of 0.25 s. For MS/MS experiments, two types of fragmentation techniques (i.e., product ion and precursor ion) were performed [41]. In product ion MS/MS analysis, after selecting the specific precursor ion in the first stage, the characterizing fragment masses were scanned from *m*/*z* 50 to the upper *m*/*z* value that covers the precursor ions with an accumulation time of 0.03 s. For precursor ion scanning, precursor mass spectra were acquired in the first mass analyzer by selecting a specific product ion. The data acquisition and processing were released with SCIEX software Analyst TF™ 1.5.1.

MS spectra were analyzed, searching the lipid subclasses in the LipidMaps Database (http://www.lipidmaps.org accessed on 26 July 2023). The identification of lipid species was confirmed by molecular ions and their specific fragments with a mass tolerance of 0.2 Da, guaranteeing elimination of any possible false-positive identification.

#### 3.2.6. Performance Evaluation of Extraction Procedures

Data from SCIEX software Analyst TF™ 1.5.1. were exported in GraphPad Prism 5.0 H software for further data processing and analysis. The intra-assay precision of each protocol, as assessed by a triplicate analytical run of same sample, was expressed by standard deviation percentage (SD%) as a result of the mean of peak intensities. The inter-assay reproducibility was defined as the coefficient of variation (CV%), calculated by dividing the standard deviation of the intensities of each SPL subclass (for each method) by the relative mean, expressed as a percentage. A lower CV% corresponds to the consistency of the values obtained by the extraction of replicates.

For each extraction procedure, the percentage recovery of single standard was calculated as the ratio of the peak area of standard in a sample spiked before extraction, divided by the peak area of standard in the sample spiked at the same level after extraction. For this purpose, cell samples were spiked before and after extraction with 10 mL of a standard solution of S1P, Cer, and SM (100 ng mL^−1^ in MeOH). To obtain the peak intensities of a single standard, the extracts were analyzed by tandem mass-spectrometry in precursor ion scanning mode by selecting a product ion at *m*/*z* 78.8, at *m*/*z* 264.4, and at *m*/*z* 184.4 for S1P, Cer, and SM, respectively.

## 4. Conclusions

Modern lipidomic require the identification of the complete profile of lipid species present in a tissue, organelle, or cell, and therefore require an evaluation of possible alterations (metabolic differences) within lipid classes and subclasses, in response to physiological, pathological, and environmental conditions.

Sphingolipid studies clearly require reliable analytical methods with a high degree of specificity for the identification of molecular species structurally similar and rapidly interconverted.

The present study conducted on the hepatocellular carcinoma cell line (HepG2) led to the selection of a method for the analysis of all the key components of the sphingolipid metabolic pathway. The detection and identification of multiple sphingolipids in a single sample were made possible by combining a classic extraction technique (B&D method) with tandem mass spectrometry-shotgun (ESI-MS/MS).

The verified method proved to be a valuable analytical tool useful for studying the response of HepG2 cells to the most common anticancer physical therapy (radiotherapy). The resulting altered profile of sphingolipids confirmed that they are complex bioactive molecules, enigmatic in their pro-death and pro-growth signals in tumoral tissues. Increasingly, they can represent new therapeutic targets for the development of new antitumor intervention strategies.

## Figures and Tables

**Table 1:** Identification of SPL species in negative-ion ESI-MS/MS spectra of HepG2 extracts.

Sphingolipids	*m*/*z* Molecular Ion	CE ^1^	*m*/*z* Product Ions
Hydroxysphingosine	311.3–313.3	−35	
Methylsphinganine	323.2–325.4	−35	
Dimethylsphingosine	339.4	−35	
C17 S1P (d17:1)	367.5	−30	367.5 → 250.3, 78.8
C17 Sa1P (d17:1)	369.3	−25	369.3 → 252.2, 78.8
C18 S1P (d18:1)	381.6	−30	381.6 → 266.2, 78.8
AcylSa	409.3	−35	409.3 → 264.4
C17 S1-PC	451.3–459.3	−45	
Cer(d14:1/15:0)	473.3	−40	473.3 → 237.1
Cer(d18:1/13:0)	493.5	−40	493.5 → 264.4
Cer(d18:1/16:0)	535.5	−45	535.5 → 264.4
Cer(d18:1/19:0)	577.7	−45	577.7 → 264.4
Cer(d18:2/22:0)	619.6	−45	619.6 → 264.4
Cer(d18:1/25:0)	661.6	−45	661.6 → 264.4
GlcCer(C16:0)	703.7	−45	703.7 → 266.4
DH Cer1P(d18:0/24:0)	731.9	−45	731.9 → 266.3, 78.8
Cer1P(d18:0/25:0)	745.6	−45	745.6 → 78.9
SM(d18:0/22:0)	787.8	−40	787.8 → 184.4
SM(d18:1/26:0)	829.9	−40	829.9 → 184.4
LacCer (C16:0)	861.6	−45	861.6 → 266.2, 342.1
GlcSph	885.4	−40	
GlcSph	891.3	−40	

^1^ CE: collision energy (V).

**Table 2:** Identification of SPL species in positive-ion ESI-MS/MS spectra of HepG2 extracts.

Sphingolipids	*m*/*z* Molecular Ion	CE ^1^	*m*/*z* Product Ions
So (d14:0)	237.1	20	
So (d17:1)	281.0	20	
Sa (d19:0)	319.0	45	319.0 → 268.3
C16 Sa1P	356.6	25	356.6 →252.2, 78.8
C18 S1P (d18:1)	385.4	30	385.4 →266.2, 78.8
AcylSa	413.9	30	413.9 → 264.4
Sa1-PC	469.3	30	
Cer(d18:1/14:0)	513.9	45	513.9 → 264.4
Cer(d18:1/17:0)	559.0	45	559.0 → 264.4
Cer(d18:1/21:3)	603.9	45	603.9 → 264.4
Cer(d18:1/24:1)	647.3	45	647.3 → 264.4
Cer1P(d18:1/19:0)	666.6	45	666.6 → 264.4, 78.9
Cer(d18:1/26:1)	690.0	45	690.0 → 264.4
Cer1P(d18:0/24:0)	732.5	45	732.5 → 266.3, 78.9
Cer1P(d18:1/26:0)	758.7	45	758.7 → 264.4, 78.9
SM(d17:1/22:0)	772.3	45	772.3 → 184.4
SM(d18:1/22:0)	785.9	45	785.9 → 184.4
SM(d18:1/24:1)	811.1	45	811.1 → 184.4
LacCer (d16:0/16:0)	837.3	45	837.3 → 266.2, 342.1

^1^ CE: collision energy (V).

## Data Availability

Data are available on request.

## Supplementary Materials

The following supporting information can be downloaded at: https://www.mdpi.com/article/10.3390/ijms241512364/s1.
