# Supplementary material for: A Sphingolipidomic Profiling Approach for Comparing X-ray-Exposed and Unexposed HepG2 Cells
Source: Int J Mol Sci. 2023 Aug 2;24(15):12364. doi: 10.3390/ijms241512364 (PMC10418425; doi:10.3390/ijms241512364)
Supplement: Supplementary file 1 [file ijms-24-12364-s001.zip › ijms-2513634-supplementary.pdf]

Table S1. Assignments of FTIR bands of HepG2 cells lipid extracts according to Refs. Talari 2017, Movasachi 2008 and Dreissing 2009

**Wavenumber** (cm^{-1})	**Assignments**	**Indication**
3402 - 3360	N-H stretching	Amide A
3018 - 3009	CH olefinic stretching	Unsaturated fatty acids
2956 - 2954	CH_3 asymmetric stretching	Saturated fatty acids
2922 - 2918	CH_2 asymmetric stretching	Saturated fatty acids
2852 -2851	CH_2 symmetric stretching	Saturated fatty acids
1741 - 1735	C=O stretching	Phospholipids
1690 - 1600	C=O stretching	Sphingolipids
1553 - 1530	N-H bending	Sphingolipids
1467 - 1467	CH_2 bending	Fatty acids
1412 - 1413	CH_2 cyclic bending	Cholesterol<br>Cholesterol ester
1377 - 1353	CH_3 bending	Fatty acids
1230 - 1222	PO_2^- asymmetric stretching	Phospholipids
1181 - 1172	C–O asymmetric stretching	Phospholipids<br>Cholesterol ester
1087 - 1083	PO_2^- symmetric stretching	Phospholipids
1064 - 1051	C–O–H stretching	Sphingolipids<br>Cholesterol
1035 - 1020	C–O–P stretching	Phospholipids

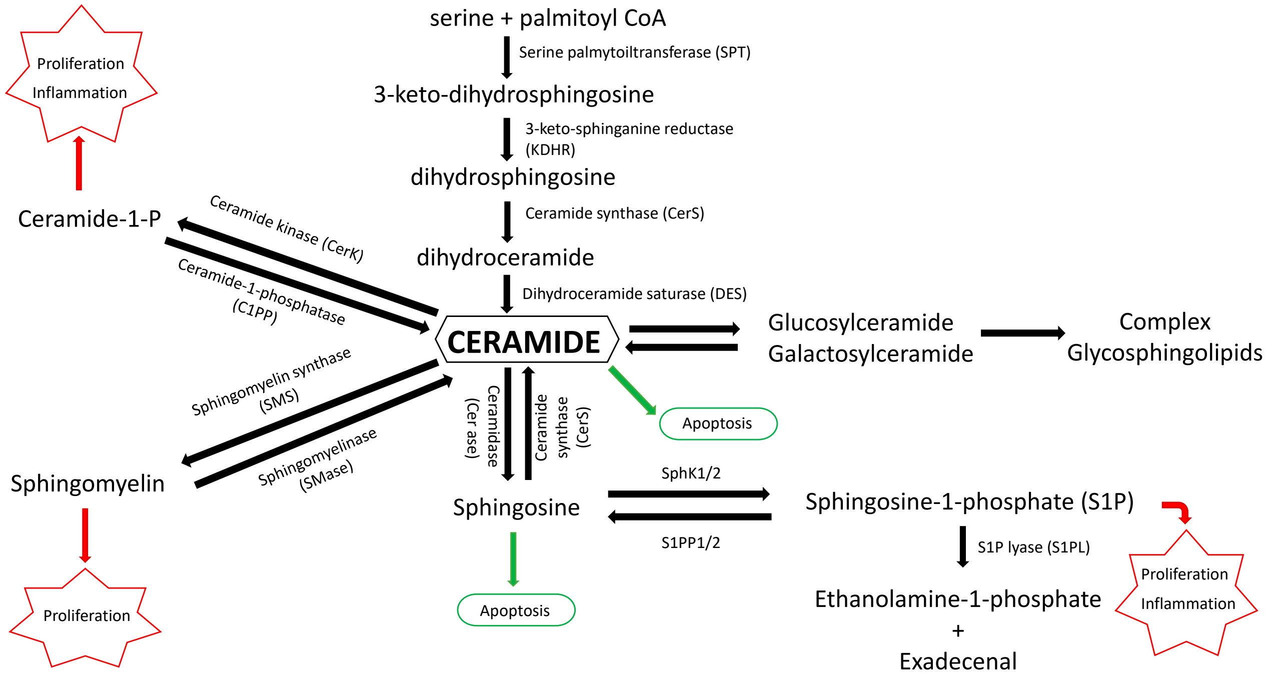

Figure S1. Schematic representation of sphingolipid metabolic network.

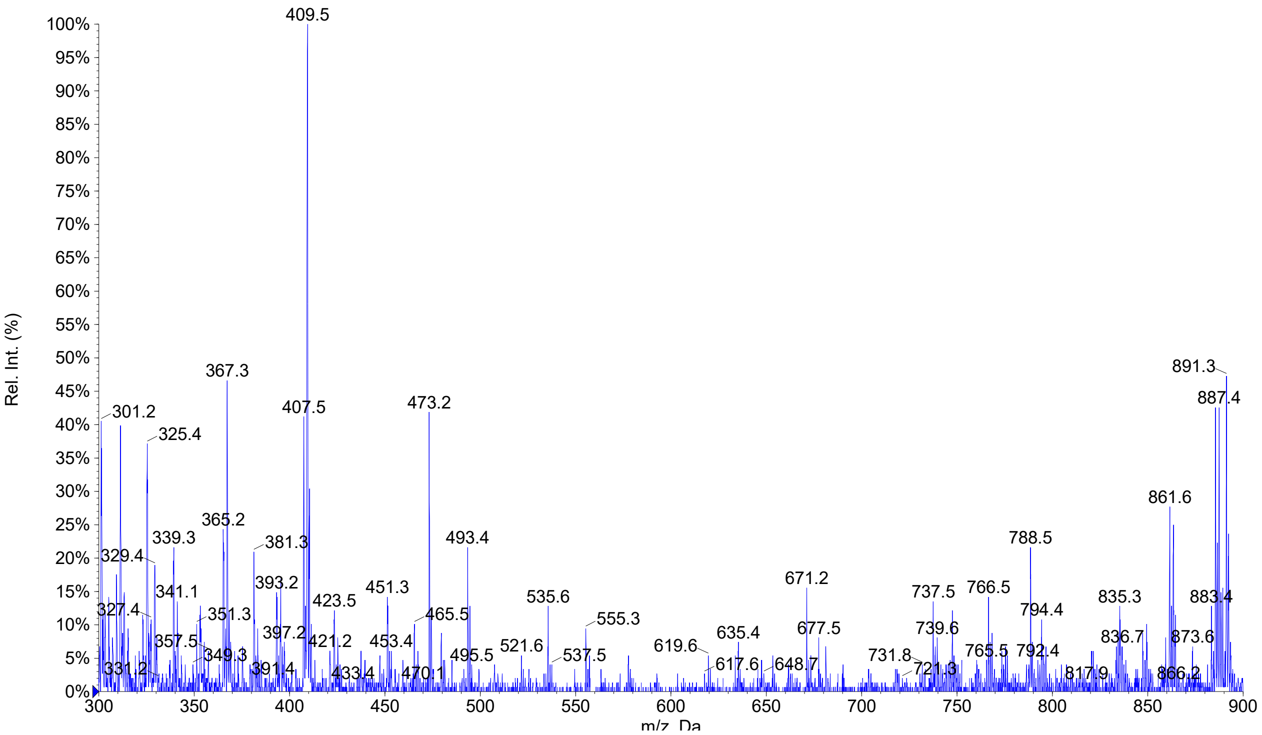

(a)

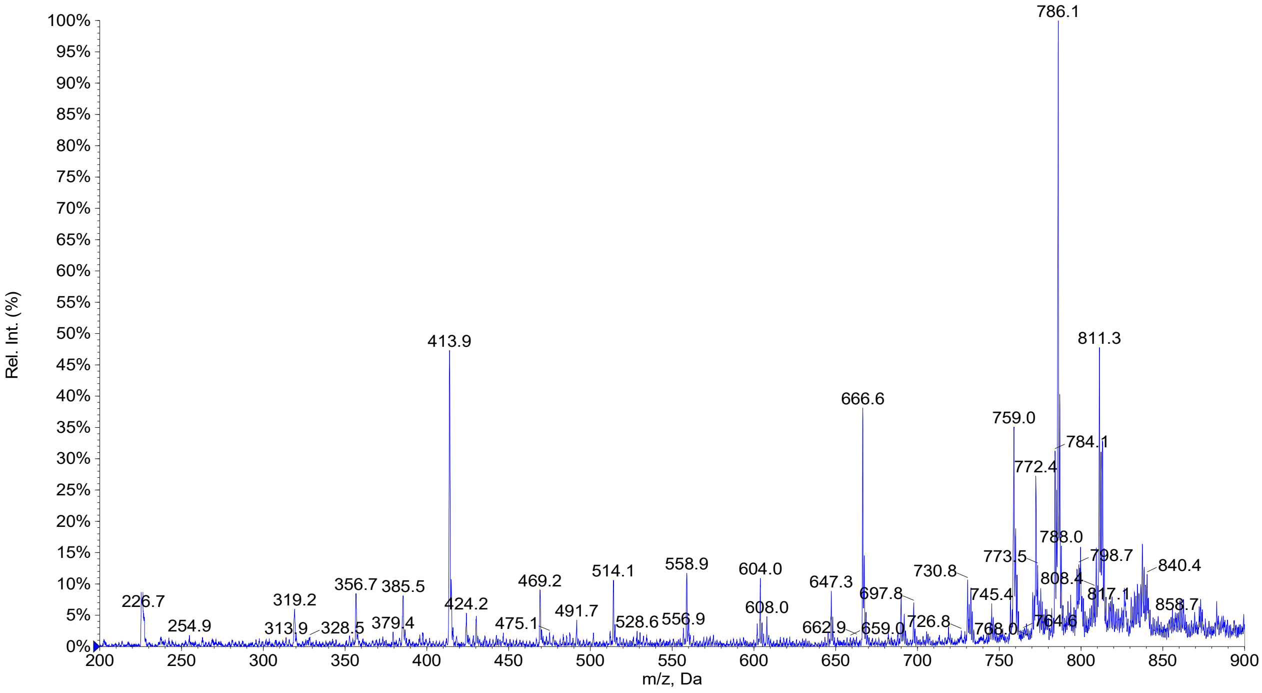

(b)

Figure S2. Representative mass spectra of samples obtained by BuMe modified method using unirradiated HepG2 cells: (**a**) in negative mode; (**b**) in positive mode.

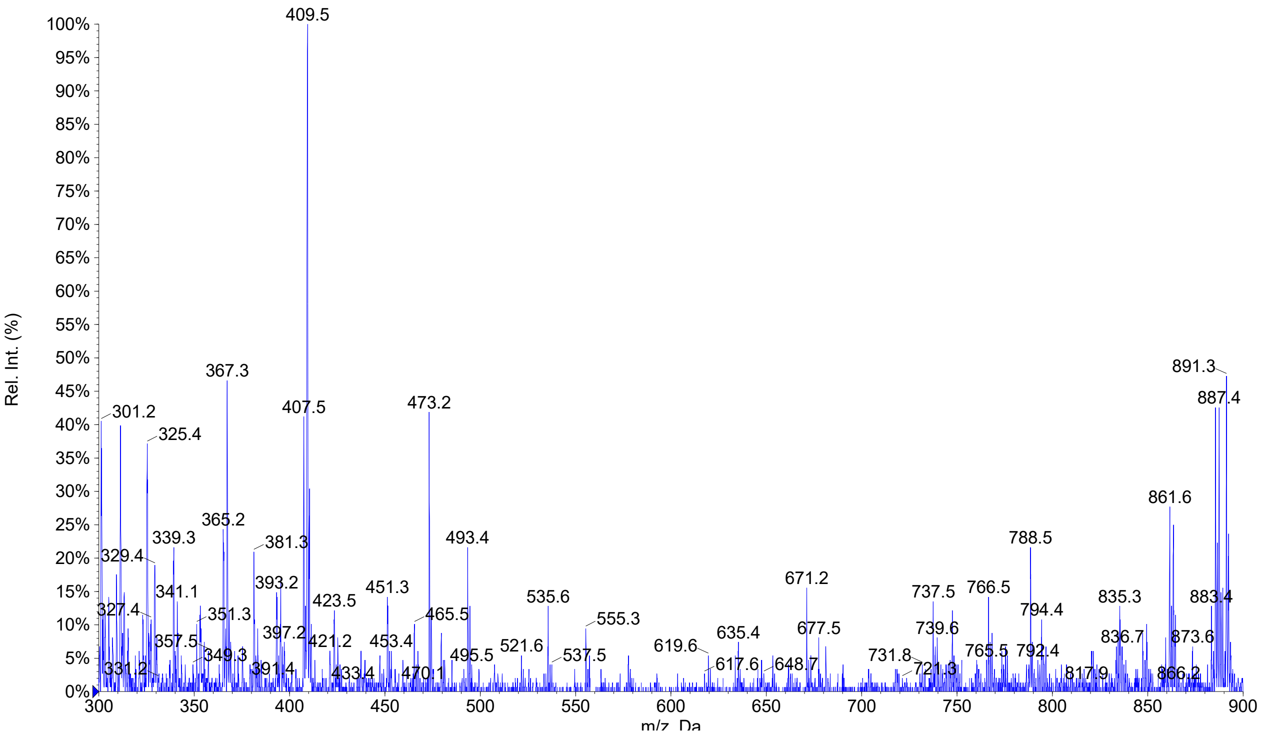

(a)

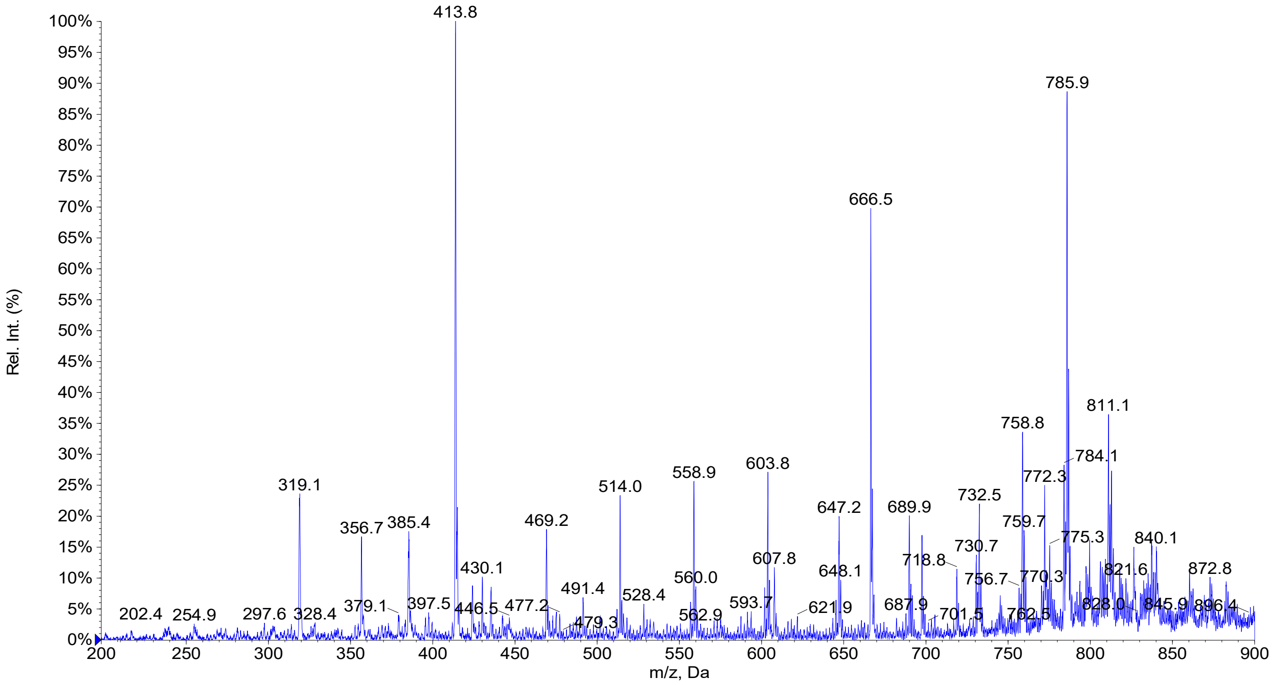

(b)

Figure S3. Representative mass spectra of samples obtained by SHA method using unirradiated HepG2 cells: (**a**) in negative mode; (**b**) in positive mode.

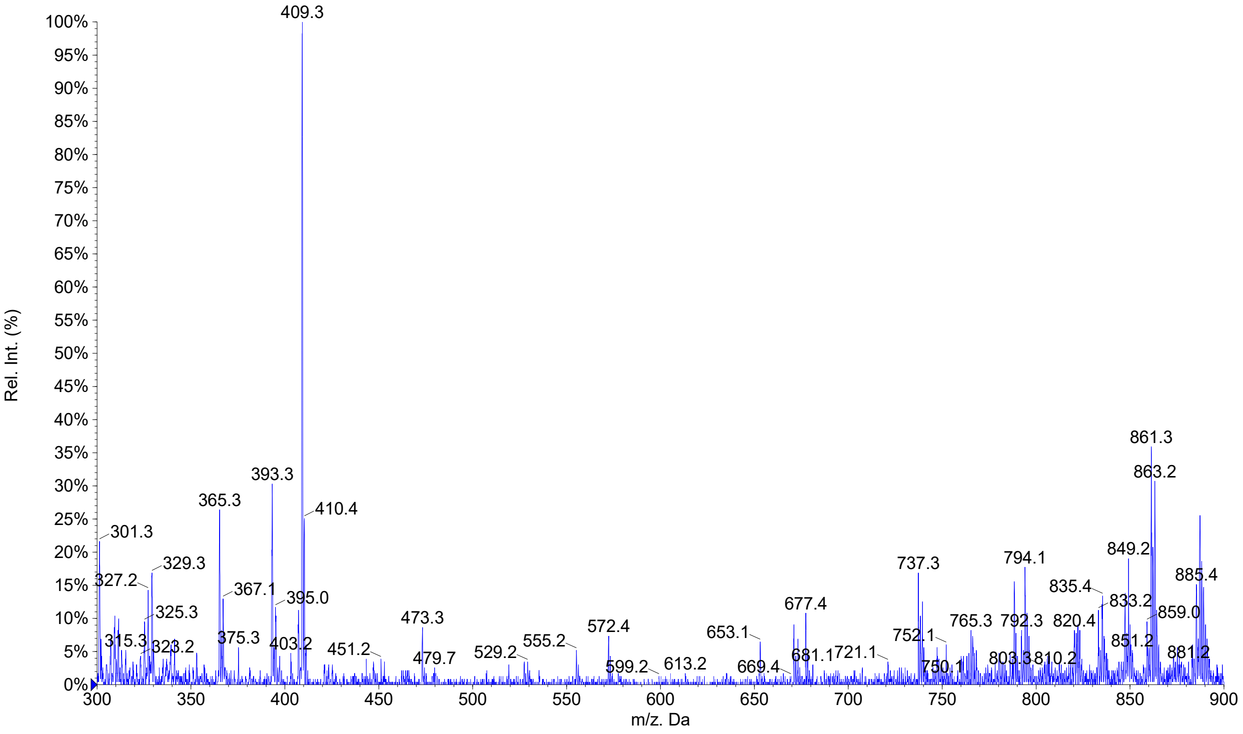

(a)

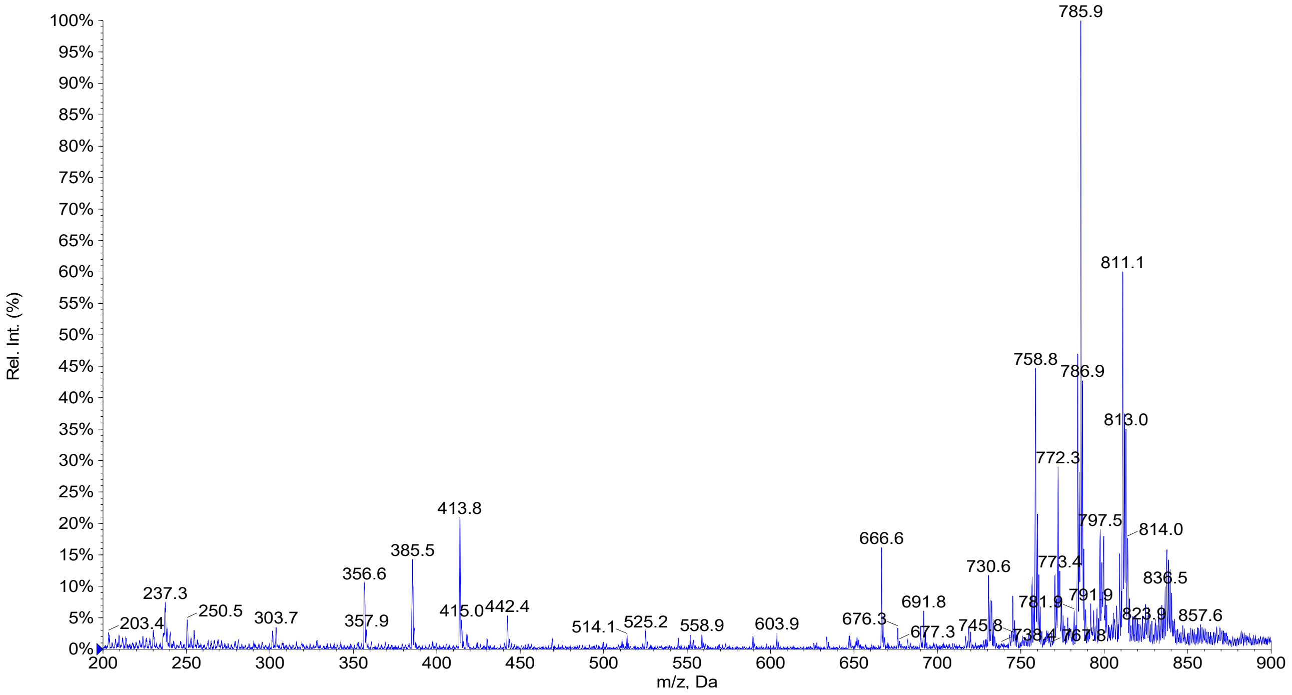

(b)

Figure S4. Representative mass spectra of samples obtained by IPA method using unirradiated HepG2 cells: (**a**) in negative mode; (**b**) in positive mode.
